# Severe pediatric COVID-19: a review from the clinical and immunopathophysiological perspectives

**DOI:** 10.1007/s12519-023-00790-y

**Published:** 2024-02-06

**Authors:** Yi-Kan Sun, Can Wang, Pei-Quan Lin, Lei Hu, Jing Ye, Zhi-Gang Gao, Ru Lin, Hao-Min Li, Qiang Shu, Li-Su Huang, Lin-Hua Tan

**Affiliations:** 1 Children’s Hospital, Zhejiang University School of Medicine, Hangzhou, 310052 China; 2 The Second Affiliated Hospital, Zhejiang University School of Medicine, Hangzhou, 310030 China; 3 Surgical Intensive Care Unit, Children’s Hospital, Zhejiang University School of Medicine, Hangzhou, 310052 China; 4 Department of General Surgery, Children’s Hospital, Zhejiang University School of Medicine, Hangzhou, 310052 China; 5 Department of Cardiopulmonary and Extracorporeal Life Support, Children’s Hospital, Zhejiang University School of Medicine, Hangzhou, 310052 China; 6 Clinical Data Center, Children’s Hospital, Zhejiang University School of Medicine, Hangzhou, 310052 China; 7 Department of Cardiac Surgery, Children’s Hospital, Zhejiang University School of Medicine, Hangzhou, 310052 China; 8 National Clinical Research Center for Child Health, Children’s Hospital, Zhejiang University School of Medicine, Hangzhou, 310052 China; 9 Department of Infectious Diseases, Children’s Hospital, Zhejiang University School of Medicine, Hangzhou, 310052 China

**Keywords:** Immunopathophysiology, MIS-C, Pediatric critical care, Severe pediatric COVID-19

## Abstract

**Background:**

Coronavirus disease 2019 (COVID-19) tends to have mild presentations in children. However, severe and critical cases do arise in the pediatric population with debilitating systemic impacts and can be fatal at times, meriting further attention from clinicians. Meanwhile, the intricate interactions between the pathogen virulence factors and host defense mechanisms are believed to play indispensable roles in severe COVID-19 pathophysiology but remain incompletely understood.

**Data sources:**

A comprehensive literature review was conducted for pertinent publications by reviewers independently using the PubMed, Embase, and Wanfang databases. Searched keywords included “COVID-19 in children”, “severe pediatric COVID-19”, and “critical illness in children with COVID-19”.

**Results:**

Risks of developing severe COVID-19 in children escalate with increasing numbers of co-morbidities and an unvaccinated status. Acute respiratory distress stress and necrotizing pneumonia are prominent pulmonary manifestations, while various forms of cardiovascular and neurological involvement may also be seen. Multiple immunological processes are implicated in the host response to COVID-19 including the type I interferon and inflammasome pathways, whose dysregulation in severe and critical diseases translates into adverse clinical manifestations. Multisystem inflammatory syndrome in children (MIS-C), a potentially life-threatening immune-mediated condition chronologically associated with COVID-19 exposure, denotes another scientific and clinical conundrum that exemplifies the complexity of pediatric immunity. Despite the considerable dissimilarities between the pediatric and adult immune systems, clinical trials dedicated to children are lacking and current management recommendations are largely adapted from adult guidelines.

**Conclusions:**

Severe pediatric COVID-19 can affect multiple organ systems. The dysregulated immune pathways in severe COVID-19 shape the disease course, epitomize the vast functional diversity of the pediatric immune system and highlight the immunophenotypical differences between children and adults. Consequently, further research may be warranted to adequately address them in pediatric-specific clinical practice guidelines.

## Introduction

Contrary to most recognized respiratory pathogens, severe acute respiratory syndrome coronavirus 2 (SARS-CoV-2), the causative pathogen of COVID-19, typically leads to milder disease in pediatric cases than in adult patients [1, 2], but young children, particularly infants, may still be prone to contract the virus [3, 4], and a small number may develop severe or even critical illnesses [5, 6]. Children with severe COVID-19 may develop serious complications such as acute respiratory distress syndrome, myocarditis, acute renal failure, cardiogenic or septic shock, and multiorgan failure, and mortality can occur in extreme cases [7]. A nationwide surveillance study conducted in the United States during the peak of the pandemic recorded a COVID-19-related hospitalization rate of 48.2 per 100,000 population for children under 18 years of age from October 2020 to September 2021, of whom 26.4% required ICU admission, 6.2% required invasive mechanical ventilation, and 0.7% died while hospitalized [8]. The epidemiology of pediatric COVID-19 has also evolved substantially since the advent of successful vaccines, with new cases aggregating mostly in unvaccinated children or subgroups of children who are ineligible for immunization [9]. The picture is further complicated by the emergence of multisystem inflammatory syndrome in children (MIS-C), a distinctive post-infectious entity that accounted for a significant proportion of pediatric ICU admissions linked to COVID-19, and highlights the profound involvement of host immune response in the pathogenesis of severe pediatric COVID-19 and the associated conditions [10–13]. This review, therefore, aims to provide a holistic dissection of the diseases of interest with focuses on both the clinical and immunological standpoints.

## Clinical approaches to severe pediatric COVID-19

### Risk factors for developing severe COVID-19 in children and red flags for deterioration

The symptomology of clinically evident acute COVID-19 in children is similar to that in adults, which mostly involves the respiratory tract, with the most common presenting complaints being fever, coughs, coryzal symptoms including nasal congestion and rhinorrhea, and dyspnea, which may be accompanied by headaches, myalgia, generalized malaise, and possibly gastrointestinal symptoms such as nausea, vomiting, decreased oral intake, and diarrhea [14–16]. Identification and close monitoring of children at risk of severe COVID-19 represent a concrete first step in clinical assessment. The risk factors with the highest relative risks for severe COVID-19 in children are chronic lung diseases, obesity, diabetes, cardiovascular disease, neurological comorbidities including seizure disorders, and prematurity (among children below 2 years of age) [1, 17, 18], many of which are linked to endothelial impairment and a pro-inflammatory state [19], and odds of critical care admission and mortality increase in a step-wise manner with increased number of comorbidities [20].

Meanwhile, it is important to clarify the vaccination status in light of the well-rounded protective effects it offers against disease transmission, progression, and complications. For instance, a recent meta-analysis of 51 studies revealed that 2 doses of mRNA vaccines are 75.3% and 78% effective against COVID-19-associated hospitalizations and MIS-C, respectively, in children between the age of 5 and 11 years [21]. However, waning protection with the emergence of novel variants (e.g., Omicron)[22] and elapsed time since the last administered dose [23], especially for those aged 5–11 years as opposed to older children or adolescents [24, 25], need to be taken into consideration. It is worth noting that immunocompromised children, even if appropriately vaccinated as per the modified schedule, are still deemed at high risk of progression to severe COVID-19 due to lower response rates to vaccinations and vulnerability to SARS-CoV-2 pathogenicity [26].

Any clinical, biochemical, and radiological signs indicating deterioration, especially in at-risk children, should be promptly acknowledged and actioned upon, which include (1) increased respiratory rate; (2) poor responsiveness, drowsiness, and convulsions; (3) lymphopenia and/or thrombocytopenia; (4) hypo/hyperglycemia and/or hyperlactatemia; (5) markedly elevated inflammatory markers such as procalcitonin, C-reactive protein, and ferritin; (6) significant transaminitis and creatine kinase elevation; (7) pronounced abnormalities in coagulation function parameters; and (8) changes in head imaging such as cerebral edema or significant progression of pulmonary lesions on chest imaging [27].

### Clinical characteristics of severe and critical pediatric COVID-19

#### Case definition

The case definition for severe pediatric COVID-19 may have slight variations across studies and guidelines, but it typically requires (1) a form of diagnostic certainty with a positive RT-PCR result for SARS-CoV-2 nucleic acids as the gold standard; (2) hospitalization as a result of COVID-19 related symptoms, thereby excluding cases managed in the outpatient setting; and (3) ICU admission, invasive mechanical ventilation, or circulatory support as the key indicators of disease severity, albeit inevitably limited by disparities in intervention thresholds between different centers and patient subgroups [1, 20]. Besides, deviations in respiratory and oxygenation indices have been used to delineate severe disease, including a blood oxygen saturation level (SpO_2_) of < 94% on room air under atmospheric pressure at sea level [27, 28] and significant elevations in age-adjusted respiratory rate [27], while conditions suggestive of other organ system dysfunctions are also proxy measures [10, 29].

#### Respiratory

The rate of viral pneumonia is determined to be 24% among children hospitalized with a COVID-19 diagnosis by a multi-center study in the US [30]. Necrotizing pneumonia (NP) is a disastrous complication of severe pediatric COVID-19, which stemmed from aggressive bacterial superinfections causing lung tissue liquefaction and cavitation, with the most common causative organisms being *Staphylococcus aureus*, *Streptococcus pneumoniae*, and *Mycoplasma pneumoniae* [31]. Specifically, Akuamoah-Boateng et al. reported the case of a 13-year-old boy with COVID-19 and convincing laboratory and radiologic features of NP presumably caused by *Prevotella oris*, which was not detected by the blood cultures prior to antimicrobial administration but returned positive on PCR of the surgical drainage sample of concurrent subdural empyemas [32]; and Brisca et al. reported a case of NP in a 4-month-old infant with of COVID-19 and concurrent central venous catheter-associated methicillin-susceptible *Staphylococcus aureus* bacteremia [33]. NP in both cases eventually resolved with appropriate level of respiratory support and multimodal pharmacotherapy.

Physicians should also be kept aware of COVID-19-related croup [34] that has shown increased incidences with Omicron variants [35, 36], manifests as vocal hoarseness, stridor, wheezing, or lung rales [34, 37, 38], and in critical cases may require endotracheal intubation and cardiopulmonary resuscitation, although COVID-19-associated croup is treated similarly to other viral causes of croup and respiratory distress is generally uncommon [39].

Additionally, a scrutinization of the Overcoming COVID-19 registry data has shown that acute respiratory distress syndrome (ARDS) was seen in approximately 10% of hospitalized children with severe acute COVID-19 and MIS-C [10], compared to 33% in admitted adults [40]. Despite lower incidences compared to adults, COVID-19-related pediatric ARDS required mechanical ventilation in most cases [41, 42], and the strategy of low tidal volume and limiting plateau pressure for lung protection has been routinely implemented [41, 43–45]. ARDS was also found to be independently associated with lower probability of discharge from PICU and hospital in a multivariable analysis of time to discharge [41], which reflects its protracted disease course and risk of lethality.

#### Cardiovascular

**Fig. 1:**
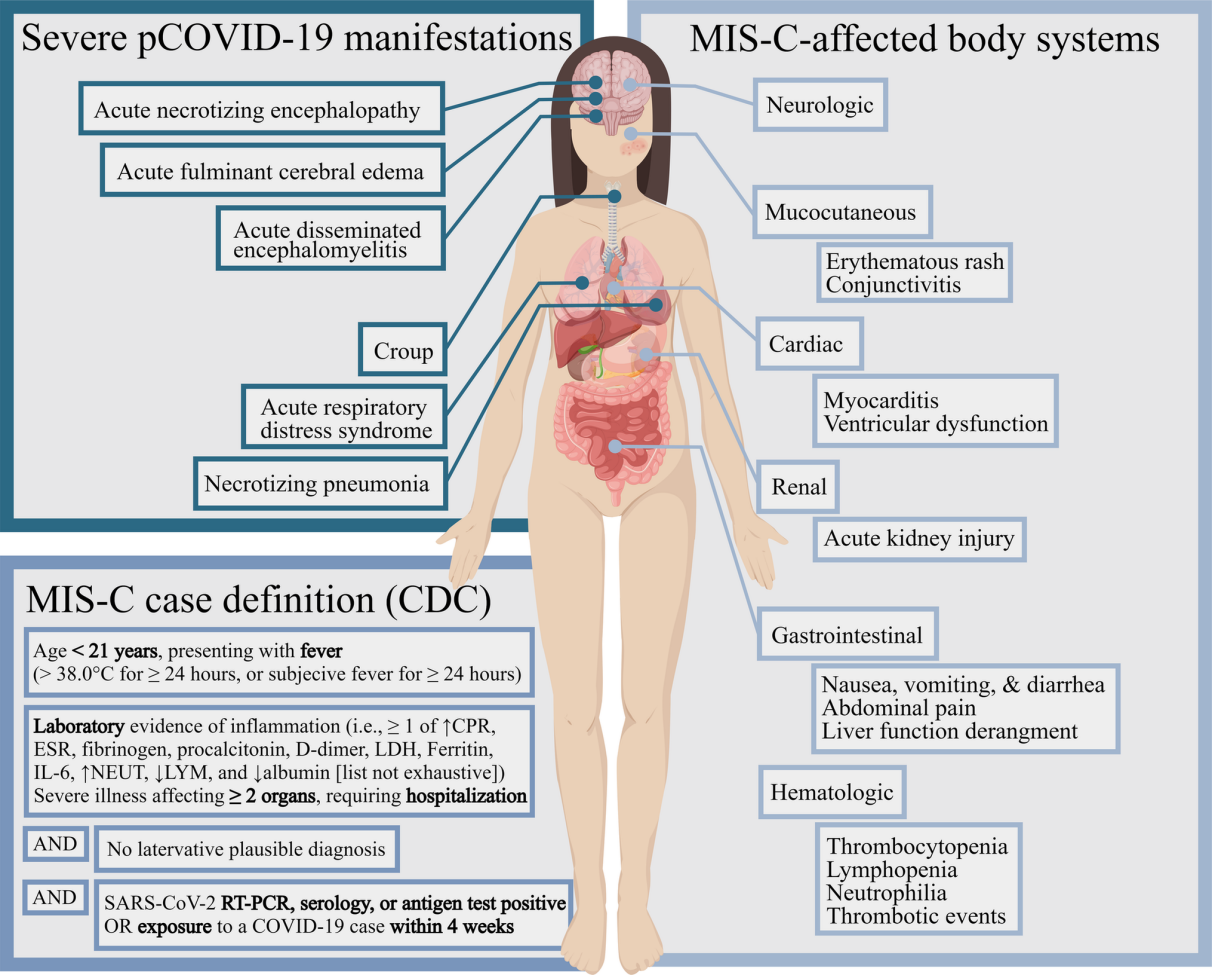
Potentially life-threatening manifestations of severe pediatric COVID-19 and organ systems implicated in MIS-C. The MIS-C diagnostic criteria are adapted from the Centers of Disease Control and Prevention of the United States. *pCOVID-19* pediatric COVID-19, *CRP* C-reactive protein, *ESR* erythrocyte sedimentation rate, *LDH* lactate dehydrogenase, *IL-6* interleukin 6, *NEUT* neutrophils, *LYM* lymphocytes, *RT-PCR* reverse transcription polymerase chain reaction

Cardiogenic shock [46], pericarditis [47], and myocardial injury [48–50] have all been reported in acute SARS-CoV-2 infection in children, and evidence of persistent cardiac injury could be detected 3–6 months on cardiac magnetic resonance imaging after severe pediatric COVID-19 [51]. However, a large proportion of COVID-19-related cardiovascular involvement in pediatric patients may be driven by indirect, non-cardiac insults, especially MIS-C [52]. 93% of the 283 children with MIS-C in a multicenter European study had myocardial injury, as reflected by elevated troponin levels [53], while reduced ejection fraction can be seen in approximately 30% of MIS-C patients [10, 53]. Even though SARS-CoV-2 RNA was detected with myocarditis on post-mortem cardiac biopsy of an 11-year-old child who died of MIS-C, suggesting direct viral invasion could be the inciting and perpetrating event in this particular case [54], a systemic inflammatory response is likely the principal driving force for myocarditis in most MIS-C cases [53, 55], which is known for its multiorgan manifestations (Fig. 1) and will be discussed in further details in connection with an evaluation of its elusive pathogenesis.

#### Neurologic

Non-specific neurologic symptoms are relatively common in children with COVID-19, whereas serious neurologic manifestations are much more infrequent, with an estimated prevalence of 3.8% among pediatric patients admitted to hospital with COVID-19 [56]. A significant proportion of them may be broadly categorized as neuroimmune disorders, among which numerous cases of acute disseminated encephalomyelitis [57–61], a demyelinating disease affecting the central nervous system, as well as Guillain–Barré syndrome [62, 63] have been reported. On the other hand, direct viral invasion of the central nervous system (CNS) by SARS-CoV-2 is rare but has been described [64], and disruption of the blood–brain barrier and host immunity secondary to COVID-19 may predispose patients to lethal CNS co-infections, including opportunistic ones by *Mycobacterium Tuberculosis* [61]. Furthermore, cytokine storm and systematic inflammation may be the driving force of life-threatening neurologic conditions seen in severe pediatric COVID-19 such as acute necrotizing encephalopathy, a para-infectious condition most commonly precipitated by viruses of the *Orthomyxoviridae* family that is characterized by multifocal symmetrical lesions, especially in bilateral thalami [65, 66], and acute fulminant cerebral edema, which can happen in previously healthy children, causing brain herniation and death within 24–48 hours of foretelling seizures [67, 68]. Moreover, a well-conducted US study utilizing surveillance data showed that 47% of the pediatric patients hospitalized with COVID-19-related illnesses who developed life-threatening neurologic conditions met the diagnostic criteria for MIS-C [11].

## Pathogenic and immunologic determinants of disease severity

### SARS-CoV-2 cellular binding and entry

**Fig. 2:**
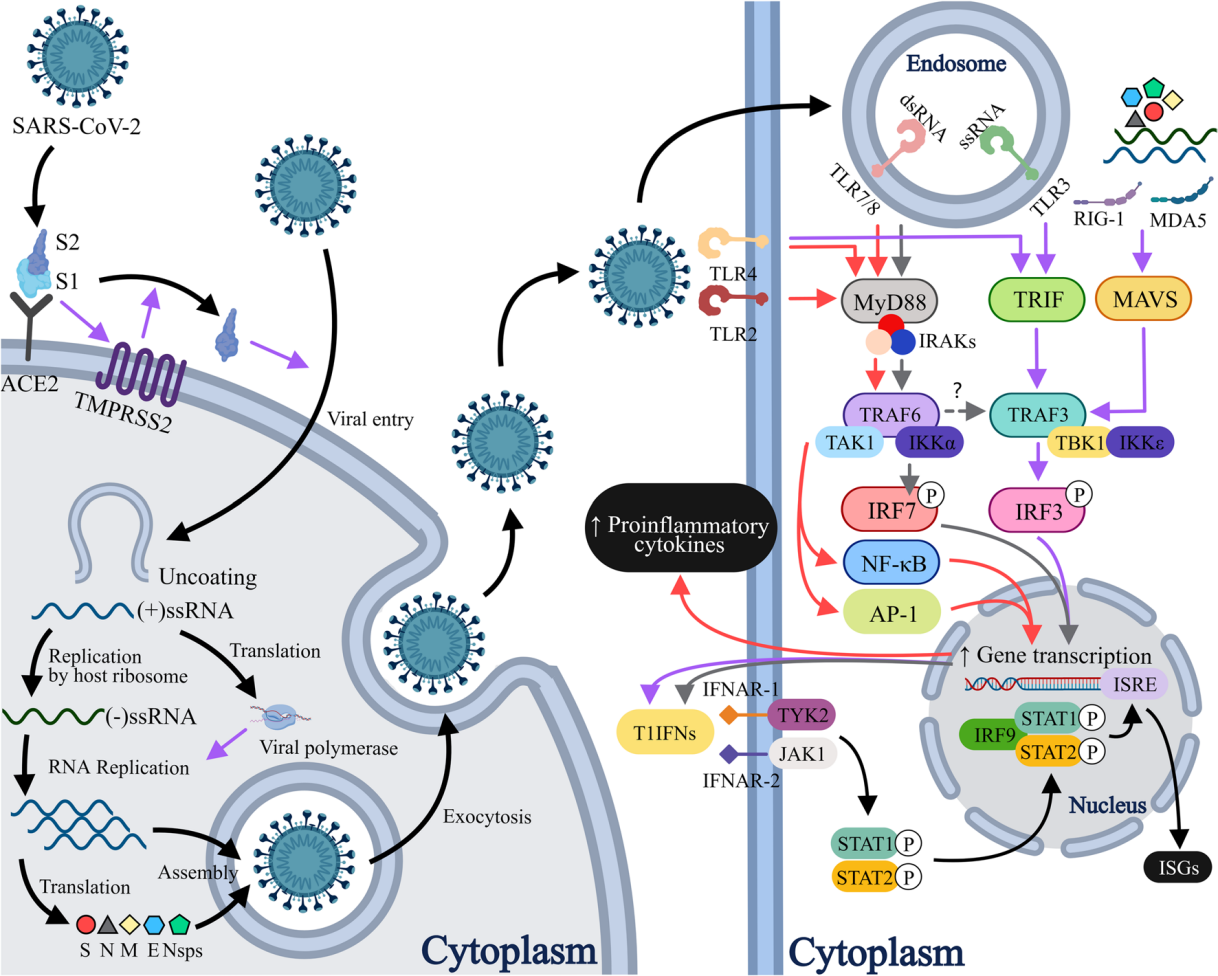
ACE-2-TMPRSS2-mediated SARS-CoV-2 cell entry and type 1 interferon pathway. SARS-CoV-2-associated molecules are recognized by a wide range of PRRs located in different compartments, including TLR2, 4 and 6 on the plasma membrane, TLR3, 7 and 8 in endosomes, and RIG-1 and MDA-5 in the cytoplasm, each with its respective ligands. Ligand binding causes TLRs to dimerize and instigate the downstream signaling pathways in an MyD88-dependent or TRIF-dependent manner. Activation of TLR2, 4, 7, and 8 recruits the canonical adaptor protein MyD88, which sequentially mobilizes the IRAK complex, TRAF6, and TAK1. TAK1 is then capable of initiating the IKK-NFκB and the MAPK-AP1 pathways, stimulating production of various proinflammatory cytokines. In addition, activation of TLR7 or TLR8 also triggers IRAK, TRAF6, TRAF3, and IKKα-dependent phosphorylation and thus activation of IRF7. Contrarily, TRAF3 may be activated by TRIF recruitment following TLR3 and TLR4 activation, or MAVS recruitment secondary to RIG-1 or MDA5 activation. TRAF3 in turn gives rise to TBK1 and IKKε activation that potentiates IRF3. Both IRF3 and IRF7 act as transcription factors that promote T1IFN gene expression. T1IFNs bind to the heterodimeric IFNAR1/IFNAR2 receptor complex, which triggers the receptor-associated kinases TYK2 and JAK1 to phosphorylate STAT1 and STAT2 proteins. The phosphorylated STAT1 and STAT2 combine with IRF9 to form the ISGF3, which binds to IRSE in the nucleus to upregulate transcription of ISGs, exerting multitudinous antiviral effects. *PPRs* pattern recognition receptors, *ACE2* angiotensin-converting enzyme 2, *TMPRSS2* transmembrane serine protease 2, *(+)/(–)ssRNA* positive-/negative-sense single-stranded ribonucleic acid, *S* spike protein, *N* nucleocapsid protein, *M* membrane protein, *E* envelop protein, *Nsps* non-structural proteins, *dsRNA* double-stranded ribonucleic acid, *TLR* toll-like receptor, *RIG-1* retinoic acid-inducible gene I, *MDA5* melanoma differentiation-associated protein 5, *MyD88* myeloid differentiation primary response factor 88, *IRAKs* interleukin-1 receptor-associated kinases, *TRIF* toll-interleukin-1 receptor-domain-containing adaptor-inducing interferon-‍β, *MAVS* mitochondrial antiviral signaling protein, *TRAF* tumor necrosis factor receptor-associated factor, *TAK1* transforming growth factor-β activated kinase 1, *IKK* inhibitor of nuclear factor-κB (IκB) kinase, *TBK1* TANK-binding kinase 1, *IRF* interferon regulatory factor, *NF-κB* nuclear factor kappa B, *MAPK* mitogen-activated protein kinase, *AP-1* activator protein 1, *T1IFNs* type 1 interferons, *IFNAR* interferon-alpha receptor, *TYK2* tyrosine kinase 2, *JAK1* Janus kinase, *STAT* signal transducer and activator of transcription, *ISGF3* interferon-stimulated gene factor 3, *ISRE* interferon-sensitive response element

Like SARS-CoV, SARS-CoV-2 utilizes its spike (S) glycoprotein to facilitate entry into target cells via interaction with the angiotensin converting enzyme II (ACE2). The S1 subunit of the S protein binds to ACE2, which activates an accompanying host protease, most commonly transmembrane serine protease 2 (TMPRSS2), releasing the S2 subunit from the S1-S2 complex [69–72]. The S2 subunit then enables fusion of the viral envelope with the cellular membrane and consequently endocytosis of the viral components [73] (Fig. 2). SARS-CoV-2 cellular tropism is therefore largely determined by co-expression of ACE2 and TMPRSS2, which is present in cells from multiple tissue origins, including nasal secretory and ciliated cells [74], alveolar epithelial type II cells (AT2s) [75], enterocytes of the small and large intestines [76, 77], proximal tubular cells of the kidney [78], cardiomyocytes [79], and vascular endothelial cells [78, 80–82], suggesting clinical manifestations in the respective organ systems may be at least partially attributed to direct cellular invasion and damage. Though still debated, many have argued that relatively lower expression and distinct distribution pattern of these entry factors in infants and children may confer protection against severe disease [83–86].

### Innate immunity as the first line of defense that arbitrates the course of disease

SARS-CoV-2 is encountered by a cascade of innate immune responses in vivo, which are crucial determinants of disease course in children [87–90]. Apart from ACE2 binding, the S protein, along with other viral constituents, may act as pathogen-associated molecular patterns (PAMPs) to activate pattern recognition receptors (PRRs), which drives the production of an array of cytokines that govern responses following the viral infection. Specifically, SARS-CoV-2 proteins, single-stranded genomic RNA, and double-stranded RNA (dsRNA) replication intermediates are sensed by various subtypes of toll-like receptor (TLRs) [91–94], and cytosolic RNA by the retinoic acid-inducible gene-I (RIG-I)-like receptors (RLRs) such as RIG-I and melanoma differentiation-associated gene 5 (MDA-5) [95], which ultimately evoke potent interferon (IFN) responses.

Induction of type I IFNs and, to a lesser extent, type III IFNs constitutes the backbone of innate immunity against SARS-CoV-2, which exerts its far-reaching effects via the JAK/STAT signaling pathway to ultimately mobilize hundreds of IFN-stimulated genes (ISGs) with a myriad of direct and indirect (i.e., via recruitment of immune cells) anti-viral functions [96, 97] (Fig. 2). Age-associated features such as more robust mucosal IFN response [89, 98], more preformed cytosolic PRRs in cells populating the upper airways [99] and more efficient RIG-1 signaling [100] in the pediatric population are believed to correlate with milder presentation of COVID-19, whereas severe disease could result from blunted type 1 IFN responses [101, 102] as a consequence of in-born defects in type 1 IFN-mediated immunity [103–107], presence of IFN-neutralizing autoantibodies [108–110], and a multitude of inhibitory factors employed by SARS-CoV-2 such as its structural and non-structural proteins and the way it replicates inside enclosed membranes to evade host immune detection [96, 111–113].

Strikingly, around 10% of children hospitalized for COVID-19 pneumonia were found to have complete recessive deficiencies in one of the four type 1 IFN immunity-related molecules [107]. Inappropriate type I/III IFN responses failing to facilitate viral clearance at the point of initial contact, most commonly the upper airway, can precipitate paradoxical hyper-inflammation with delayed but sustained release of type I IFNs down the stream and may be accompanied by unchecked upregulation of pro-inflammatory cytokines that, in turn, mediate the deleterious pulmonary and systemic inflammation seen in severe and critical illnesses [73, 88, 96, 98, 102, 111, 114, 115]. Consequently, the therapeutic values of exogenous IFN formulations for COVID-19 are subject to the timing of administration. Available clinical trials found no added benefits of IFN beta-1a in hospitalized adults [116, 117], while outpatients were protected from emergency department visits and hospital admissions with a dose of pegylated interferon lambda administered within 7 days of symptom onset [118]. Relevant pediatric data are scarce.

### Raging hyper-inflammation signifies severe COVID-19

**Fig. 3:**
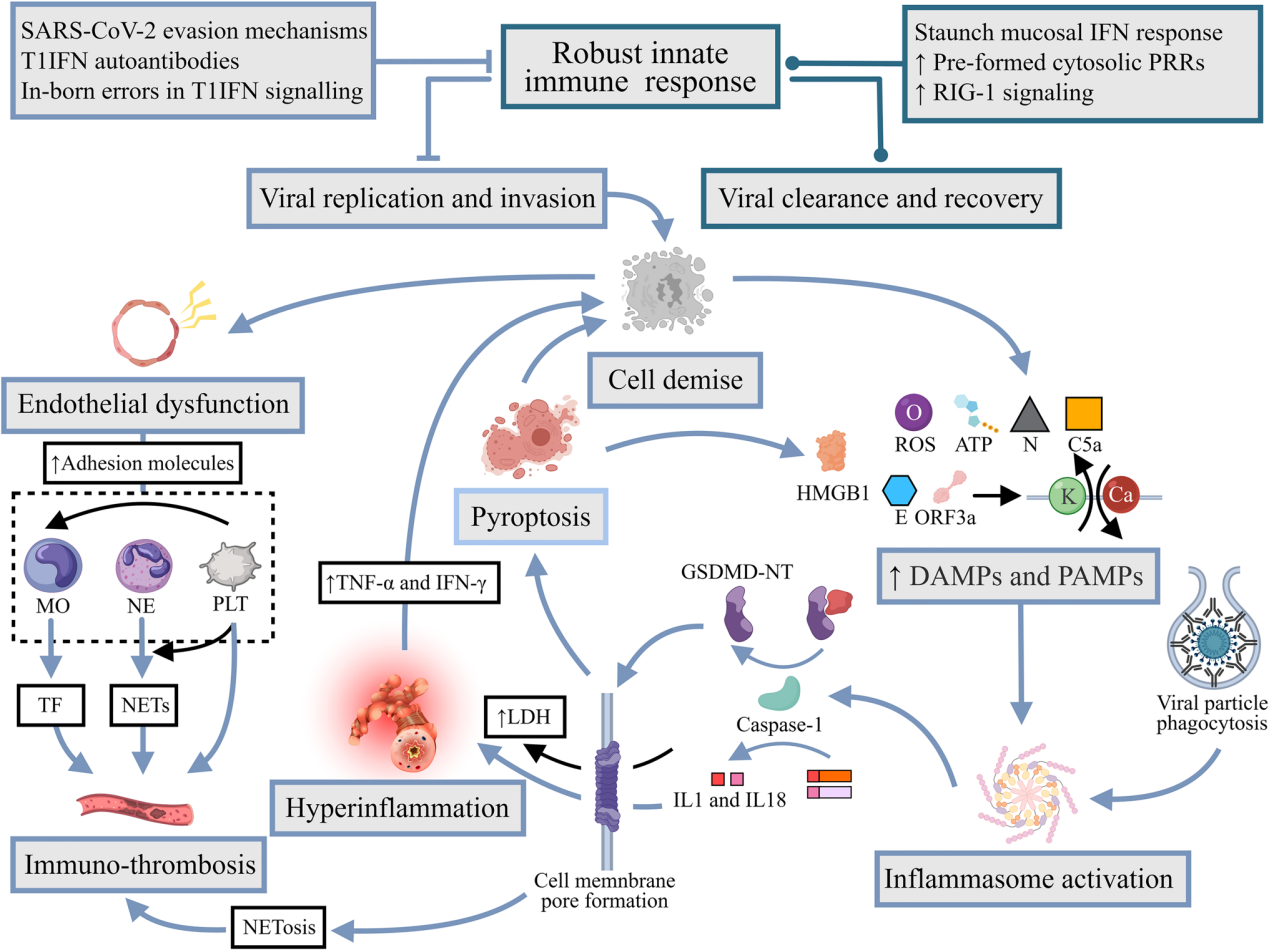
Hyperinflammation and immunothrombosis in severe COVID-19. Virus-related factors like the N protein and potassium efflux and calcium influx set off by envelope and ORF3a “viroporin” proteins, and host-related factors such as extracellular ATP, complement C5a, ROS, and phagocytosis of antibody-opsonized viral particles into otherwise ACE2^−^ monocytes have all been proposed to trigger assembly of inflammasomes, most notably NLRP3, in myeloid-derived immune cells and pulmonary cells. Inflammasome activation recruits the caspase-1 canonically, which proteolytically potentiates pro-IL-1β and/or pro-IL-18 and concomitantly cleaves GSDMD into NT and CT fragments. GSDMD-NT oligomerization and translocation to plasma membrane create pores through which activated IL-1β and IL-18 can directly enter the extracellular space, mediating hyperinflammation, and simultaneously trigger pyroptosis, leading to LDH and HMGB1 release, among other pro-inflammatory DAMPs. Meanwhile, endothelial dysfunction in the hyperinflammatory milieu initiates immunothrombosis. Adhesion molecules are markers of activated endothelial cells over-expressed in COVID-19 that exhibit strong anchoring effects on monocytes and neutrophils, the former of which reciprocate with active TFs that directly institute the extrinsic coagulation pathway. Conversely, neutrophils may be prompted by direct SARS-CoV-2 entry, SAR-CoV-2-induced ROS generation, complement activation, and/or a self-sustaining loop of IL-8 production to undergo NETosis, defined by the release of large extracellular web-like structures termed NETs, which may also be triggered by the inflammasome/GSDMD pathway in COVID-19. NETs consist of decondensed chromatin embellished with histones and proteins that act as a scaffold for erythrocyte and platelet settling and fibrin deposition, while its constituents exert a range of pro-thrombotic effects with varying mechanisms. Furthermore, circulating platelets adopt a hyperactive state in the context of SARS-CoV-2 infection. Platelets recruited in response to NETs and other stimuli such as vWF on the activated endothelial cells in turn amplify NETosis via secretion of chemokines such as PF4, and may induce further expression of TF by monocytes and complementarily augment monocytic secretion of inflammatory cytokines. *N protein* nucleocapsid protein, *ORF3a* open reading frame 3a, *ATP* adenosine triphosphate, *ROS* reactive oxygen species, *NLRP3* NOD-like receptor containing pyrin domain 3, *IL-1β* interleukin-1β, *IL-18* interleukin-18, *GSDMD* gasdermin-D, *NT* N-terminal, *CT* C-terminal, *LDH* lactate dehydrogenase, *HMGB1* high mobility group box 1, *DAMPs* damage-associated molecular patterns, *TF* transcription factor, *IL-8* interleukin-8, *NETs* neutrophil extracellular traps, *vWF* von Willebrand Factor, *PF4* platelet factor 4

Dysregulated host inflammatory response gains pathogenic dominance over the viral burden itself with COVID-19 disease progression, as evidenced by otherwise no clear correlation between viral load and disease severity, including in the pediatric population [119–121]. In severe/critical COVID-19, complex and intertwined concurrent immunopathological proceedings (Fig. 3) ensue as a result of viral dissemination under the circumstances of waning innate immunity. AT2s, which are made susceptible due to the aforementioned dual ACE2/TMPRSS2 positivity, and other cells succumbing to viral invasion release PAMPs as well as danger-associated molecular patterns (DAMPs), which are hallmarks of cellular stress that elicit various forms of programmed cell death (RCD) [122, 123]. Inflammasomes are micrometer-level multiprotein signaling complexes that form in the cytoplasm via combining particular nucleotide-binding and oligomerization domain(NOD)-like receptors, a subtype of PRRs, with the respective adaptor molecules, secondary to priming and activation by PAMPs and DAMPs [124]. Inappropriate activation of inflammasomes has been demonstrated to play indispensable roles in linking different compartments of immunity and orchestrating the hyper-inflammatory reaction to SARS-CoV-2, with the decisive endpoint being the caspase-mediated interleukin-1b and interleukin-18 release through cell membrane-spanning pores formed by gasdermin-D oligomerization [125–128] (Fig. 3). Markers of inflammasome activation were indeed found elevated proportionately to disease severity in the sera of critically ill COVID-19 patients [126]. Pyroptosis, a form of inflammatory caspase-dependent RCD evident in as many as 6% of the monocytes in the peripheral blood of COVID-19 patients [129], is another well-recognized eventuality in the context of porous plasma membrane [130] leading to release of large intracellular molecules such as lactate dehydrogenase, which is pathognomonic for pyroptosis and a laboratory parameter of prognostic value in clinical practice [131, 132], as well as further outpouring of inflammatory cytokines and DAMPs, such as high mobility group box 1 [133]. The self-propagating vicious cycle of hyper-inflammation and cell death is thus established and culminates in dire clinical consequences. Systemically, severe COVID-19 can elicit a cytokine profile somewhat similar to that of the cytokine storms secondary to other etiologies [134–136]. Synergism of cytokines such as TNF-α and IFN-γ [137] has been demonstrated to be capable of inducing inflammatory cell death and thus may inflict tissue damage directly to end-organs and perpetuate the vicious cycle.

### Features of pediatric adaptive responses in COVID-19

The traits of cellular and humoral immunity specific to children may become apparent when challenged with SARS-CoV-2. Mobilization of T and B cell pathways synchronized with the innate defense in the upper airway in children, thereby preventing severe disease development from viral dissemination [138]. Lymphopenia is a much-feared consequence and indicator of poor prognosis consistently seen in hospitalized, ICU-admitted, and non-surviving COVID-19 patients [139–141], albeit less common in children [15], that may be partly attributed to the plethora of pro-inflammatory cytokines [88, 142, 143]. Nevertheless, adults were found to have stronger CD4^+^ and CD8^+^ T cell responses in the acute infective phase, which were proposedly a compensation for the inferior innate responses and, indeed, did not lead to favorable outcomes [98, 144, 145]. By the same token, some studies have shown that SARS-CoV-2 invokes a less vigorous antibody response in children that was restricted to S protein-specific IgG production, as opposed to anti-S IgM, IgG, and IgA as well as antibody formation against other viral proteins in adults [146–148]. Otherwise, there have been mixed results regarding the neutralizing activity of SARS-CoV-2-specific immunoglobulins derived from children [144, 149, 150], although they are generally longer lasting than their adult counterparts, offering protection from re-infection for months and beyond [147, 149, 151, 152].

### Devastating cross-talk of the inflammatory and coagulation pathways

In the lungs, the highly inflammatory alveolar microenvironment is the harbinger of alveolar epithelial injury and dysfunction, supported by transcriptomic evidence of AT2 and AT1 exhaustion and demise in fatal COVID-19 [153–155]. The resultant denudation of the alveolar basement membrane and exposure of the underlying endothelial cells to the detrimental cocktail of hypoxia, viral content, apoptotic and necrotic debris, immune cells, cytokines, and chemokines trigger their activation, transformation to a leaky state due to cytoskeleton and intercellular junction alterations [156], and potentially direct cell death that, in combination with the increased epithelial permeability, precipitates leukocyte extravasation and accumulation of the proteinaceous edema characteristic of ARDS [73, 157].

In the meantime, loss of the usual quiescent endothelial cell phenotype under the influence of cytokine overdrive ignites the sophisticated and incompletely understood interplay between the immune and hemostatic mediators in an attempt to limit the spread of the pathogen that ultimately leads to the micro- and macro-thrombus formation frequently observed in severe/critical COVID-19 [158–161], as alveolar capillary microthrombi are found to be close to 10 times as prevalent in patients who died of COVID as in those who died of ARDS following H1N1 influenza [162]. The injurious positive feedback loop of immunothrombosis, which may notably involve NETosis [163, 164] (Fig. 3), eventually gives rise to the formation of the pathognomonic alveolar hyaline membranes in ARDS [157, 165]. The fibrin-rich exudates may significantly compromise the alveolar-capillary interface for gas exchange, resulting in the profound refractory hypoxemia seen in respiratory failure secondary to severe/critical COVID-19 [73, 165].

Similarly, there are evidently increased incidences of pulmonary emboli [166, 167], thrombosis, and angiopathy of the microvasculature that may contribute to multi-organ failure [168, 169], and extrapulmonary arterial and venous thromboembolic phenomena in adults [170, 171], and to a lesser degree, in children and adolescents with COVID-19, where nine (2.1%) of the 426 hospitalized pediatric patients with symptoms developed thrombotic events across seven children’s hospitals in the US [172]. Therefore, thrombo-inflammatory markers such as D-dimer are the most common abnormal laboratory findings in adult and pediatric COVID-19 [173], and may be predictive of disease course [174, 175].

### MIS-C, a riddle unresolved

Since the emergence of SARS-CoV-2, surges in MIS-C cases appear to aggregate 3–6 weeks following peak incidences of COVID-19 cases in a heavily affected locale and whose symptomology bears resemblance to but is distinct from Kawasaki disease (KD), toxic shock syndrome (TSS), and macrophage activation syndrome (MAS) [176–178] (Fig. 1). MIS-C may share clinical features of KD including sustained high fevers, conjunctivitis, diffuse non-vesicular erythematous rash, and dry and cracked lips, but clearly diverges from KD in that it (1) is more frequently reported in older children of non-Asian descent with an average age of 9 years, no apparent gender bias, and an overall mortality rate of 2%, and increased age may be associated with risks of ICU admissions [12, 13], which are required for up to 65%–70% of all cases[179–181]; (2) has a markedly high rate of gastrointestinal (GI) tract (80%) and neurological involvement [11, 182], and cardiac manifestations as discussed but less likelihood of developing severe or persistent coronary artery aneurysms [10, 53, 55, 183, 184]; and (3) is notably associated with ferritinemia in severe disease and thrombocytopenia [185, 186].

Although there are commonalities of shock, multi-organ failure, endothelial damage and coagulopathy, neutrophilia, lymphocytopenia, elevated inflammatory markers between severe/critical pediatric COVID-19 and MIS-C, the latter is less likely to have primary respiratory implications and mostly arise in otherwise healthy children and adolescents devoid of major pre-existing co-morbidities who have already seroconverted with IgG predominance at time of MIS-C diagnosis and mild to no symptoms on initial infection or exposure [187]. MIS-C also carries distinguishing cytokine signatures including enrichment of the type II interferon (IFN-γ) and the downstream effector molecules [115, 188–192] that may explain its homogeneous features with MAS. Admittedly, the immunological landscape of MIS-C appears rather complex and only partially defined, and there has not been a unifying pathophysiological blueprint to account for the manifold stigmata of MIS-C, but much progress has been made in delineating the nuances in immunophenotypes that may shed light on their similarities and differences to other systemic inflammatory syndromes.

Given the overlaps between MIS-C with TSS, which has bacterial superantigens as the unequivocal trigger [193], and detection of SARS-CoV-2 in various organs on autopsy of patients who died of MIS-C [194], it is reasonable to hypothesize that SARS-CoV-2 proteins may possess superantigenic properties that set off the hyper-inflammatory response in MIS-C, where computational analysis has indeed identified a sequence motif exclusive to SARS-CoV-2 S protein capable of T cell activation that shows striking structural similarity to a segment of the staphylococcal enterotoxin B, the culprit responsible for TSS [195, 196]. The definitive reservoir of superantigens in MIS-C, however, has not yet been found, given the varied nasopharyngeal RT-PCR positivity status in MIS-C cases [197]. In accordance with the prominent gastrointestinal complaints in MIS-C, persistence of SARS-CoV-2 in the GI tract [198, 199] and subsequently the compromise to the intestinal barrier integrity have been posited as a potential route of antigenic entry and dissemination, as evidenced by elevation of enterocyte damage [200] and intestinal permeability [201] and inflammation [202] makers in MIS-C children, although studies have yielded conflicting results on whether antigenemia is present in most cases of MIS-C [115, 201, 203]. Moreover, numerous groups have unanimously demonstrated a phenomenon typical of superantigenic stimulation where, similar to TSS, MIS-C is characterized by extensive polyclonal proliferation of a specific T cell receptor (TCR) β-chain variable domain subset, namely TRBV11-2 in MIS-C [115, 200, 201, 204–207], and de-escalation of the expansion appears to coincide with abatement in inflammatory cytokine levels and clinical improvement with therapy, especially after glucocorticoid administration [115, 205], further supporting the potential roles of TRBV11-2-expressing T cells in pathogenesis. On the contrary, many studies have detected autoantibodies against endothelial, cardiac, gastrointestinal, and immune antigens [207–210] in MIS-C as the alternative pathogenic mediators that may provoke damage to the respective organ systems, although intravenous immunoglobulin therapies may be a confounding factor [115, 211].

In addition, several genetic predispositions have been identified for MIS-C including the specific combination of HLA class I alleles A02, B35, C04 that is associated with TRBV11-2 expansion [115, 204], albeit not supported by findings of many groups [200, 205, 206] possibly due to differing ethnic compositions of the study populations, as well as flaws in down-regulators of inflammation such as an autosomal recessive defect in the OAS-Rnase L pathway, which normally disposes of the cytosolic dsRNA that can stimulate production of pro-inflammatory cytokines [212], and nonsynonymous mutations in XIAP, CYBB and SOCS1 genes [213]. Ongoing immune profiling efforts are underway to advance mechanistic understanding of this perplexing disease entity, as with many other inflammatory conditions, through which more universally applicable insights may also be generated into the broader immune system as a whole.

## Management, prognosis, and long-term sequelae

Fortunately, COVID-19 in the vast majority of children is self-limiting and would not qualify for treatment other than supportive care and symptomatic management until spontaneous resolution, and although pediatric clinical trial data are lacking, the number needed to treat for therapeutic benefits is considered to be higher in children than in adults [214].

Several pharmacological agents are available to reduce the risk of progression to severe disease in vulnerable outpatients, should the potential benefits be deemed to outweigh the risks. Paxlovid, a combination medication of nirmatrelvir and ritonavir available in oral formulations that can be conveniently taken in the outpatient setting, has been demonstrated in the cornerstone phase III trial involving 1219 adults at-risk for severe disease to have an astonishing 89% risk reduction in COVD-19-related hospitalizations or deaths if administered within 3 days of symptom onset [215]. Paxlovid appears still highly effective in the time of Omicron preeminence [216], but has only been authorized by the US Food and Drug Administration (FDA) for use in children aged 12 years or older and weighing 40 kg or greater, and is limited by its extensive interactions with other medications due to ritonavir being a potent CYP3A inhibitor [217]. Alternatively, remdesivir represents the sole FDA-authorized agent for prevention of disease progression in the community setting for at-risk and mildly to moderately symptomatic children with at least 28 days of age and a weight of at least 3 kg, with the logistical caveat of requiring intravenous dosage delivery on 3 consecutive days [218]. In contrast, clinical decisions regarding inpatient management of severe COVID-19-related diseases in children are not infrequently extrapolated from adult guidelines and made on a case-by-case basis given the scarcity of large-scale randomized controlled trials in the pediatric population from which evidence-based recommendations can be derived [16, 219, 220].

Despite the generally favorable outcomes even in the critically ill children [221], the full picture of the long-term impacts of COVID-19 on pediatric health may not be readily apparent at this stage given the recency of the pandemic. The term long COVID, sometimes also referred to as post-acute sequelae of COVID-19, has been coined as the diagnosis of exclusion to encompass the constellation of complaints after the acute stage of COVID-19 has settled [222]. One or more persistent symptoms are reported in 16.2% of children for 3 months or greater post-infection as estimated by a recently published meta-analysis [223]. Some of the most commonly reported symptoms range from persistent sore throat, fever, dyspnea, anosmia/ageusia, muscle weakness, and coughs to various vague and largely non-localizing neuropsychiatric troubles, including fatigue, mood and sleep disturbances, and mental dysfunction [223–226], seemingly so heterogeneous that categorizations in clinical phenotypes of diverse pathogenesis for subgroup analysis may be advisable [227]. Children with less than 5 years of age, underlying comorbidities, or admission to ICU during the acute phase of disease are found to be especially susceptible [228], but symptomatic or asymptomatic non-hospitalized patients are not precluded from developing the condition [223]. Of note, among the several pathophysiological hypotheses that have been put forward for long COVID are viral persistence [229], autoimmunity [230], endothelial dysfunction with microcirculation thrombosis [231, 232], and immune dysregulation with potential reactivation of latent viral infections such as Epstein-Barr virus and herpesviruses given the shared features with myalgic encephalomyelitis/chronic fatigue syndrome and dysautonomia including orthostatic intolerance and profound fatigue [233–235]. The rather limited understanding of long COVID at this point in time has prompted well-coordinated systematic research undertakings such as the RECOVER initiative to comprehensively delineate the disease, which notably has a branch of effort dedicated to children and young people [236].

## Expert opinion and conclusions

Various measures can be implemented at different stages to minimize adverse outcomes associated with severe COVID-19 in children. Vaccination proves to be effective in reducing severe disease development and should be administered as per protocol, if not contraindicated.

Red flags for deterioration should be recognized and attended to without delay, especially in children with predisposing comorbidities. Management of severe disease should generally observe a holistic approach to account for the multi-system manifestations, where emphases are placed on airway and respiratory status optimization, hemodynamic support, modulation of the detrimental hyperinflammatory response, addressing the comorbid conditions and superinfections if any, and preservation of organ functions.

The contrasting susceptibilities and responses to SARS-CoV-2 and the variations in the disease course are, to a great extent, an attestation of the monumental differences in the ways pediatric and adult immune systems are programmed to resist a highly immunogenic viral threat. In conjunction with protective antibodies with longer lifespans and lower expression of SARS-CoV-2 entry factors, a swiftly induced T1IFN response of the optimal magnitude at the mucosal surface and upper airway serves as a powerful protective barrier for children against viral spread, consequently mitigating the risk of severe COVID-19, which may see indiscriminate firing of various proinflammatory apparatuses such as inflammasomes and NETs. Further research on severe pediatric COVID-19 and relevant conditions such as MIS-C and long COVID remains of scientific and clinical significance, as it offers a novel viewpoint for deciphering the age-specific characteristics of immunity. With ongoing intermittent outbreaks, clinicians must remain vigilant of the telltale signs of deterioration, especially in those children at risk, and rationalize the use of therapeutic measures for the best outcome, although more pediatric-specific clinical trials are required before recommendations with high level of evidence can be made, as unsuspecting application of findings in adult studies to pediatric patients is a fundamentally unscientific practice that disregards the unique qualities of pediatric immunity and physiology. Additionally, rapidly evolving SARS-CoV-2 strains and the immunological memory that has formed along the way will only make it more difficult to interpret earlier studies. In more general terms, the collective knowledge gained from the COVID-19 pandemic serves as a methodological construct for better understanding of and responding to similar communicable diseases with heavy pediatric disease burdens, such as influenza and mycoplasma pneumonia, as well as existing and emerging pathogens with pandemic potential.
